# Recurrent Lymphocytic Meningitis Positive for Herpes Simplex Virus Type 2

**DOI:** 10.3201/eid1507.080716

**Published:** 2009-07

**Authors:** Katariina Kallio-Laine, Mikko Seppänen, Hannu Kautiainen, Marja-Liisa Lokki, Maija Lappalainen, Ville Valtonen, Markus Färkkilä, Eija Kalso

**Affiliations:** Helsinki University Central Hospital, Helsinki, Finland (K. Kallio-Laine, M. Seppänen, M. Lappalainen, V. Valtonen, M. Färkkilä, E. Kalso); Orton Orthopedic Hospital, Helsinki (H. Kautiainen); Haartman Institute, University of Helsinki, Helsinki (M.-L. Lokki)

**Keywords:** Meningitis, aseptic, herpes simplex virus type 2, prevalence, neurologic manifestations, viruses, Finland, dispatch

## Abstract

We found the prevalence of recurrent lymphocytic meningitis associated with herpes simplex virus type 2 (HSV-2) was 2.2/100,000 population in Finland during 1996–2006, higher than previous estimates. PCR was most sensitive in detecting HSV-2 DNA from cerebrospinal fluid if the sample was taken 2–5 days after symptom onset.

Recurrent lymphocytic meningitis (RLM) is a rare disease, characterized by attacks of sudden onset aseptic meningitis followed by complete recovery and unpredictable recurrences. The disease is most often caused by herpes simplex virus type 2 (HSV-2) and less frequently by other viruses, autoimmune disorders, or medication. Symptomatic episodes of RLM usually subside within 5 years, but the total number of episodes may reach 30. Patients are typically middle-aged, and women are more often affected than men (*1**–**3*). In addition to symptoms typical of meningitis, ≈50% of patients have transient hallucinations, seizures, cranial nerve palsies, or an altered level of consciousness (*4*).

## The Study

This study was conducted at Helsinki University Central Hospital, Finland, which serves a population of 1.4 million. The prevalence study covered January 1996 through December 2006. This period differed from that of the patient study because the World Health Organization’s coding system changed in 1996 to the International Classification of Diseases, 10th Revision. Diagnostic codes A87, B00.3 + G02.0, B01.0 + G02.0, B02.1, G02*, G03.0, G03.1 and G03.2 were used to identify study cases.

The patient study was conducted from January 1994 through December 2003. All patients with RLM (>2 clinical episodes, lymphocytic predominance and negative bacterial culture from cerebrospinal fluid [CSF], and HSV-2 DNA in at least 1 CSF sample) were recruited. A structured questionnaire was used to interview patients about symptoms during and after meningitis episodes. Antibodies against HSV types 1 and 2 were tested on the study entry day, which was at least 1 month after the most recent RLM episode. Sixty-two age- and sex-matched healthy participants served as controls in the laboratory analysis.

Type-specific HSV-1 and -2 immunoglobulin (Ig) G and IgM were measured by enzyme immunoassay (HerpeSelect 1&2 ELISA IgG; Focus Diagnostics, Cypress, CA, USA; and EIAgen HSV IgM; Adaltis, Bologna, Italy). The detection of HSV DNA in CSF samples was performed as described (*5*).

Statistical comparisons between groups were made by using a permutation test for titiers of antibodies against HSV-2, and Fisher exact test for HSV seropositivity. Kaplan-Meier estimate was used to illustrate information on the cumulative proportions of the second meningitis episode.

During the prevalence study, from January 1996 through December 2006, a total of 665 patients were treated at the Helsinki University Central Hospital for lymphocytic meningitis. Meningitis was recurrent in 37 patients (5.6%). Twenty-eight patients with RLM had HSV-2 DNA in CSF. In addition, 3 patients had recurring genital herpes and elevated HSV-2 serum titers. Thus, the minimum 11-year period prevalence of RLM was 2.7/100,000 population (95% confidence interval [CI] 1.9–3.7) and that of HSV-2 associated RLM 2.2/100,000 population. HSV-2 was the likely etiologic agent in 84% of all RLM cases. Six patients (16%) had no herpetic etiology. One had systemic lupus and 1 had Sjögren syndrome; in 4 patients, etiology remained unknown.

During the patient study, from January 1994 through December 2003, 86 patients had a CSF sample positive for HSV DNA. Of these patients, 23 (27%) were diagnosed with RLM; 22 case-patients (age: mean 40 years, range 25–55 years; 18 females, 4 males) were enrolled in the study.

HSV-1 seropositivity was less common in case-patients than in controls (25% vs. 52%; p = 0.043). All case-patients and 19% of the controls were seropositive for HSV-2 (p = 0.003). IgG antibody titers against HSV-2 were higher in case-patients than in seropositive controls (median 118 vs. 79; p = 0.034). IgM against HSV was not detected in 96% of the episodes.

The 22 case-patients had a combined 95 episodes (mean 4.3) of meningitis. The presence of HSV DNA in CSF had been analyzed during 48 episodes (Table 1). HSV-2 DNA was present in 82% of the samples taken during the first 2–5 days, and in 46% of samples obtained 24–48 hours after the first symptoms. If the sample was obtained either earlier or later, no HSV-2 DNA was detected, despite previous HSV-2 DNA-positive episodes. The median leukocyte count during the first HSV-2 PCR positive episode was 350 cells/mm^3^ (range 44–1,410 cells/mm^3^). In PCR negative cases, the leukocyte counts were lower.

**Table 1:** Presence of HSV type 2 DNA in CSF, leukocyte count, and timing of CSF samples in 22 patients with recurrent lymphocytic meningitis, through December 2007, Finland*

Patient no.	Age, y	No. episodes	Period (first episode–latest episode)	HSV-2 DNA in CSF	Leukocyte counts and timing of CSF samples in PCR-tested cases
1	29	7	2000–2004	NT, P, NP, NT, NP, NP, NP	**44**,† 18,‡ 4,‡ 2,‡ 1§
2	55	9	1985–2004	NT, NT, NT, NT, NT, P, P, NP, NP	**219**,§ **221**,§ 9,† 3†
3	44	4	1990–1998	NT, NT, NT, P	**310**§
4	43	4	1988–2000	NT, NT, NT, P	**790**§
5	33	4	1994–1998	NT, P, P, P	**1,410**,§ ¶,§ **73**§
6	32	4	1996–2002	P, NP, NP, NP	**164**,§ 64,† 32,† 93†
7	48	5	1979–2002	NT, NT, NT, P, NT	**196**§
8	33	4	1998–2005	NT, P, NP, NP	**360**,† 238,§ 239#
9	36	13	1990–2007	NT, NT, NT, NT, NT, NT, NT, NT, P, NP, NP, NP, NP	**195**,§ 2,† 0,† 260,§ 1‡
10	40	4	1990–1994	NT, NT, P, NT	**40**¶
11	47	2	2001–2003	P, NP	**1,006**,§ 790§
12	40	2	1989–1997	NT, P	**325**§
13	36	2	2001–2004	P, P	**592**,§ **620**§
14	38	2	1991–1996	NT, P	**92**§
15	51	3	1975–1998	NT, NT, P	**680**†
16	38	7	1992–2006	NT, P, NT, NP, NT, NT, P	**160**,† 16,‡ **106**§
17	51	3	1984–1998	NT, NT, P	**360**†
18	39	3	1989–2004	NT, NT, P	**172**§
19	45	5	1980–2004	NT, NT, NT, P,P	**350**,§ **203**§
20	25	2	2002–2004	P, NP	**1,350**,§ 430§
21	33	4	1995–2007	P,P, P, NT	**720**,§ **306**,§ ¶§
22	38	2	1990–1999	NT, P	**645**†

*HSV, herpes simplex virus; CSF, cerebrospinal fluid; NT, HSV DNA not tested; P, HSV-2 DNA present in CSF; NP, HSV-2 DNA not present in CSF. **Boldface** indicates HSV-2 DNA present in the sample. †CNF sample taken 24–48 h after first symptoms. ‡CSF sample taken within 24 h of the first symptoms. §CSF sample taken 2–5 days after first symptoms. ¶Leukocyte count or timing of the sample not known. #CSF sample taken >5 days after first symptoms.

The median patient follow-up time was 16.2 years (range 4–32 years). The number of meningitis episodes per case-patient varied from 2 to 13 (Table 1), and the number of meningitis episodes per follow-up year was 0.28 (95% CI 0.22–0.35). The time between the first and the second episode of meningitis ranged from 1 to 216 months (median 47 months; Figure).

**Figure:**
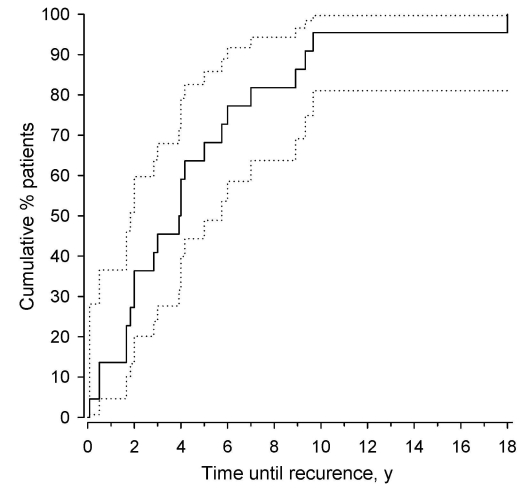
Kaplan-Meier curve showing cumulative proportions of the second recurrent lymphocytic meningitis episode (the time from the first episode to the first recurrence, years) in patients, Finland. 95% confidence intervals were obtained by bias-corrected bootstrapping.

Eight case-patients (36%) reported paresthesias and 7 (32%) had neuropathic pain during or after meningitis (Table 2). The pain typically radiated to the extremities, followed a dermatome pattern, and could last up to several years after meningitis. Arthralgias (n = 6) and urinary dysfunction (n = 5) were common.

**Table 2:** Clinical symptoms in 22 patients with recurring herpes simplex virus (HSV) type 2 meningitis, during study period (January 1994–December 2003), Finland

Associated symptoms	No. patients with symptom			Maximum duration
	During meningitis episode	After meningitis episode	Total*	
Neuropathic pain	5	6	7	Years
Paresthesias	8	6	8	Months
Urinating difficulty	3	3	5	Months
Arthralgias	1	6	6	Months
Paresis of facial nerve	1	1	2	Weeks
Hallucinations	1	0	1	Days
Dysarthria	3	0	3	Days
Visual disturbance	2	0	2	Days

*Some patients experienced symptoms both during and after episodes.

In 56 (61%) of the 95 episodes, case-patients received antiviral medication to alleviate symptoms. Seven case-patients (32%) used daily antiviral medication to prevent new episodes; 2 experienced new episodes despite the medication.

Five case-patients (23%) experienced their first episode of meningitis after symptomatic HSV-2 genital herpes; 4 of these case-patients were HSV-1 seronegative at that time. In 4 case-patients (18%), genital herpes recurred frequently (more than 6 episodes per year). Four case-patients (18%) had a history of labial herpes; 7 (32%) had no history of herpetic infections.

## Conclusions

The prevalence of HSV-2–associated RLM has been estimated to be 1/100,000 population (*6*). According to our study, the 11-year prevalence of HSV-2–associated RLM was higher, 2.2/100,000 population. Because the disease is only periodically active with long asymptomatic periods, its accurate prevalence in the population is difficult to define.

Paresthesias, neuropathic pain, arthralgias, and urinary dysfunction were common during and after meningitis. Case reports have described paresthesias associated with meningitis (*7**,**8*). Radiculomyelitis, urinary retention, and neuralgia associated with HSV-2 genital herpes have been reported, but rarely in connection with RLM (*9**–**11*).

The case-patients in our study were less often seropositive for HSV-1, and their IgG titers against HSV-2 were higher than in controls. Lately, the seroprevalence of HSV-1 has declined in developed countries, while that of HSV-2 has increased. Complications after the disease may be more frequent in HSV-2 infections without preceding HSV-1 infection (*12*).

In an earlier study, during a 1-year follow-up, recurrent disease developed in 18% of patients with HSV-2 DNA in CSF (*11*). In our study, during the 10-year follow-up, the percentage of case-patients with recurrent disease was 27% of all HSV-2 DNA-positive cases.

In RLM, all episodes are most likely caused by the same etiologic agent. However, HSV-2 DNA is not always found in CSF, despite earlier HSV-2 DNA–positive episodes. The viral load and leukocyte counts reach higher levels during the first episode of meningitis compared with recurrent cases (*13*). False-negative results may be due to a lower viral load or to earlier timing of the CSF sample in recurrent episodes (*14*). In our study, most PCR-positive samples were taken 2–5 days after the onset of acute symptoms, which is considered to be optimal timing. Clinical symptoms were alleviated in recurrent episodes, probably because of milder inflammatory changes in the CNS and lower viral load, which may result in PCR becoming less sensitive in diagnosis.

HSV-2–associated RLM is more common than previously reported. Prophylactic antiviral therapy may have decreased the incidence of recurrences but was not universally effective. In case-patients with frequent relapses, the recovery was prolonged, and residual symptoms were common.
